# A High-Throughput Interbacterial Competition Screen Identifies ClpAP in Enhancing Recipient Susceptibility to Type VI Secretion System-Mediated Attack by *Agrobacterium tumefaciens*

**DOI:** 10.3389/fmicb.2019.03077

**Published:** 2020-02-05

**Authors:** Hsiao-Han Lin, Manda Yu, Manoj Kumar Sriramoju, Shang-Te Danny Hsu, Chi-Te Liu, Erh-Min Lai

**Affiliations:** ^1^Institute of Plant and Microbial Biology, Academia Sinica, Taipei, Taiwan; ^2^Institute of Biotechnology, National Taiwan University, Taipei, Taiwan; ^3^Institute of Biological Chemistry, Academia Sinica, Taipei, Taiwan; ^4^Agricultural Biotechnology Research Center, Academia Sinica, Taipei, Taiwan

**Keywords:** type VI secretion system, antibacterial activity, recipient cells, ClpP, ClpA, *Agrobacterium tumefaciens*, *Escherichia coli*

## Abstract

The type VI secretion system (T6SS) is an effector delivery system used by Gram-negative bacteria to kill other bacteria or eukaryotic hosts to gain fitness. The plant pathogen *Agrobacterium tumefaciens* utilizes its T6SS to kill other bacteria, such as *Escherichia coli*. We observed that the *A. tumefaciens* T6SS-dependent killing outcome differs when using different T6SS-lacking, K-12 *E. coli* strains as a recipient cell. Thus, we hypothesized that the *A. tumefaciens* T6SS killing outcome not only relies on the T6SS activity of the attacker cells but also depends on the recipient cells. Here, we developed a high-throughput interbacterial competition platform to test the hypothesis by screening for mutants with reduced killing outcomes caused by *A. tumefaciens* strain C58. Among the 3,909 strains in the *E. coli* Keio library screened, 16 mutants with less susceptibility to *A. tumefaciens* C58 T6SS-dependent killing were identified, and four of them were validated by complementation test. Among the four, the *clpP* encoding ClpP protease, which is universal and highly conserved in both prokaryotes and eukaryotic organelles, was selected for further characterizations. We demonstrated that ClpP is responsible for enhancing susceptibility to the T6SS killing. Because ClpP protease depends on other adapter proteins such as ClpA and ClpX for substrate recognition, further mutant studies followed by complementation tests were carried out to reveal that ClpP-associated AAA^+^ ATPase ClpA, but not ClpX, is involved in enhancing susceptibility to *A. tumefaciens* T6SS killing. Moreover, functional and biochemical studies of various ClpP amino acid substitution variants provided evidence that ClpA–ClpP interaction is critical in enhancing susceptibility to the T6SS killing. This study highlights the importance of recipient factors in determining the outcome of the T6SS killing and shows the universal ClpP protease as a novel recipient factor hijacked by the T6SS of *A. tumefaciens*.

## Introduction

Bacteria have evolved broad strategies in secreting antibiotics or protein toxins to antagonize other bacteria and gain fitness to fight for limited nutrients and space. Among them, the Gram-negative bacteria use a variety of protein secretion systems such as type I secretion system (T1SS) (García-Bayona et al., 2017, 2019), type IV secretion system (T4SS) (Souza et al., 2015; Bayer-Santos et al., 2019), contact-dependent inhibition (CDI; belongs to type V secretion system) (Aoki et al., 2005, 2010), and type VI secretion system (T6SS) (LeRoux et al., 2012; Basler et al., 2013) as antibacterial weapons. Bacteria that produce and deliver protein toxins, the effectors, through secretion systems to kill other bacteria are attacker cells, and the attacked cells are the recipient cells (Costa et al., 2015; Filloux and Sagfors, 2015). Attacker cells also produce cognate immunity proteins to neutralize effectors to prevent self-intoxication (Alteri and Mobley, 2016; Lien and Lai, 2017). A recipient cell is intoxicated if it does not have cognate immunity protein to neutralize the toxicity of its effector.

In the CDI system, non-immunity proteins in the recipient cell also participate in the bacterial competition outcome (Aoki et al., 2008, 2010; Diner et al., 2012; Willett et al., 2015; Jones et al., 2017). For example, the CDI effector CdiA-CT^EC93^ utilizes recipient’s outer membrane protein BamA and the inner membrane protein AcrB to enter the recipient cell (Aoki et al., 2008). BamA belongs to the BAM complex that functions in outer membrane β-barrel proteins (OMPs) biogenesis. AcrB is an inner membrane protein that belongs to the multidrug efflux pump TolC complex. Another example is the necessity of the recipient *O*-acetylserine sulfhydrylase A (CysK) to the CDI effector CdiA-CT^EC536^ (Diner et al., 2012). In the recipient cell, CdiA-CT^EC536^ binds to CysK to increase its thermostability and its tRNase activity (Johnson et al., 2016). Interestingly, this CysK.CdiA-CT^EC536^ complex mimics the CysK.CysE complex, which is typically formed during *de novo* cysteine biogenesis, with a higher binding affinity (Johnson et al., 2016; Jones et al., 2017). Other examples are the recipient elongation factor Tu (EF-Tu) in activating the toxicity of CdiA-CT^EC869^ and CdiA-CT^NC101^ (Jones et al., 2017; Michalska et al., 2017), and the involvement of recipient PtsG in CdiA-CT^3006^ and CdiA-CT^NC101^ entry (Willett et al., 2015). To summarize, a variety of the non-immunity proteins in the recipient cells affect the CDI antagonizing outcome. As the bacterial secretion systems that serve as an antibacterial weapon share some universal characters, the above phenomenon raised a question of whether non-immunity proteins of the recipient cells also affect the bacterial antagonizing outcome in other secretion systems.

Recently, examples about the involvement of the recipient non-immunity proteins in T6SS competition outcome emerged. The first description is the involvement of the EF-Tu protein of the recipient cell for Tse6 effector-mediated killing by *Pseudomonas aeruginosa* (Whitney et al., 2015). Although recipient’s EF-Tu was initially proposed to grant access of Tse6 into the recipient cytoplasm (Whitney et al., 2015), a further study demonstrated that Tse6 could penetrate the double bilayer of the EF-Tu-free liposome and exert its toxicity inside it (Quentin et al., 2018). The role of the recipient EF-Tu involved in an interbacterial competition of Tse6 remains elusive. A T6SS study in *Serratia marcescens* demonstrated that the recipient protein DsbA plays a role in activating *S. marcescens* T6SS effectors Ssp2 and Ssp4, but not Rhs2 (Mariano et al., 2018). The *S. marcescens* T6SS kills its Ssp2-sensitive siblings only when the recipient cells harbor *dsbA* homologs (*dsbA1*^+^ *dsbA2*^+^). The same results were also observed using Ssp4-sensitive recipient cells, but not Rhs2-sensitive strain as a recipient cell. The above findings highlight the necessity of a recipient factor to facilitate the T6SS attack. However, a systematic screening of the recipient factors that can either promote or reduce the susceptibility of the T6SS attack is still lacking.

This study aimed to explore the recipient genetic factors that affect the T6SS killing outcome using the well-characterized T6SS-possessing plant pathogen *Agrobacterium tumefaciens*, a causative agent of crown gall disease in many different plants. The *A. tumefaciens* strain C58 harbors three effector proteins: type VI DNase effector 1 (Tde1), Tde2, and putative type VI amidase effector (Tae). The Tde proteins are the main contributor to *A. tumefaciens* T6SS-dependent interbacterial competition (Ma et al., 2014). Using the T6SS-lacking *Escherichia coli* K12 strain as a model recipient cell, we report here a high-throughput, population level, interbacterial competition screening platform for identifying the recipient genetic factors that contribute to *A. tumefaciens* C58 T6SS’s killing outcome. Among the 3,909 *E. coli* Keio mutants screened, we confirmed that at least six of them play a role in enhancing susceptibility to *A. tumefaciens* T6SS attack by an interbacterial competition assay and by complementation *in trans*. One of the confirmed genes, *caseinolytic protease P* (*clpP*), was highlighted in this study owing to its prominent phenotype. A functional ClpP complex consists of a tetradodecameric ClpP and its associated AAA^+^ ATPase substrate-recognizing partner ClpA or ClpX (Olivares et al., 2015). Further mutant studies showed that *clpA*, but not *clpX*, is involved in the outcome of *A. tumefaciens* T6SS killing. Our data also suggest that the ClpAP complex formation mediates the outcome of T6SS killing. This work not only provides a new screening platform for elucidating factors that are involved in the interbacterial competition but also strengthens the importance of recipient genetic factors in the outcome of the T6SS antibacterial activity.

## Materials and Methods

### Bacterial Strains, Plasmids, and Growth Conditions

The complete information about the strains and plasmids used in this study is described in Table 1. The *E. coli* Keio mutants (Baba et al., 2006) and the BW25113 wild type were obtained from the Keio collection from NBRP (NIG, Japan) and used as the recipient cells unless otherwise indicated. *A. tumefaciens* C58 wild type and the *tssL* mutants (Δ*tssL*) were used as the attacker cells. *A. tumefaciens* was grown at 25°C in 523 medium, and *E. coli* was grown in lysogeny broth (LB) medium at 37°C unless indicated. The plasmids were maintained in 20 μg/ml of kanamycin (Km), 100 μg/ml of spectinomycin (Sp), and 20 μg/ml of gentamycin for *E. coli*.

**TABLE 1:** Bacterial strains and plasmids.

Strain/plasmid	Relevant characteristics	Source/references
*Agrobacterium tumefaciens*		
C58 (EML530)	Wild-type virulence strain containing pTiC58 and pAtC58	Eugene Nester
C58:△*atu4333* (EML1073)	*atu4333* (*tssL*) in-frame deletion mutant of C58 background	(Ma et al., 2012)
*Escherichia coli*		
DH10B	Host for DNA cloning	Invitrogen
BW25113	Wild-type strain of the Keio Collection. *rrnB* DE*lacZ*4787 *HsdR*514 DE(*araBAD*)567 DE(*rhaBAD*)568 *rph*-1.	(Baba et al., 2006)
Keio collection	Systematic single-gene knockout mutants of *E. coli* BW25113	(Baba et al., 2006)
JW0427	BW25113 *clpP*:*kan*	(Baba et al., 2006)
JW0866	BW25113 *clpA*:*kan*	(Baba et al., 2006)
JW0428	BW25113 *clpX*:*kan*	(Baba et al., 2006)
EML5395	DH10B harboring pNptII	This study
EML5393	BW25113 wild-type harboring pNptII	This study
BL21(DE3)	Host for protein expression	(Studier and Moffatt, 1986)
**Plasmids**		
pTrc200HA	Sp^R^, pTrc200 harboring C-terminal influenza hemagglutinin (HA) epitope, P*trc*, lacI^q^, pVS1 origin	Laboratory collection
pRL662	Gm^R^, a non-transferable broad-host range vector derived from pBBR1MCS2	(Vergunst et al., 2000)
pET22b(+)	Ap^R^, *E. coli* overexpression vector harboring C-terminal 6xHis epitope	Novagen
pRL-*rpsL*	Gm^R^, pRL662 expressing BW25113 *rpsL* gene	This study
pRL-*galk*	Gm^R^, pRL662 expressing BW25113 *galK* gene	This study
pRL-*nupG*	Gm^R^, pRL662 expressing BW25113 *nupG* gene	This study
pRL-*rpsL*^Str^	Gm^R^, pRL662 expressing DH10B *rpsL*^Str^ gene	This study
pNptII	Km^R^, Gm^R^, pRL662 expressing *nptII* gene	This study
pClpP-HA	Sp^R^, pTrc200HA expressing ClpP-HA fusion protein	This study
pClpA-HA	Sp^R^, pTrc200HA expressing ClpA-HA fusion protein	This study
pClpP_S__111__A_-HA	Sp^R^, pTrc200HA expressing ClpP-HA fusion protein with S111A substitution	This study
pClpP_H__136__A_-HA	Sp^R^, pTrc200HA expressing ClpP-HA fusion protein with H136A substitution	This study
pClpP_D__185__A_-HA	Sp^R^, pTrc200HA expressing ClpP-HA fusion protein with D185A substitution	This study
pClpP_R__26__A_-HA	Sp^R^, pTrc200HA expressing ClpP-HA fusion protein with R26A substitution	This study
pClpP_D__32__A_-HA	Sp^R^, pTrc200HA expressing ClpP-HA fusion protein with D32A substitution	This study
pClpX-ΔN-ter	Plasmid used for purifying ClpX-ΔN	Robert T. Sauer
pGFP-ssrA	Plasmid used for purifying GFP-ssrA	Robert T. Sauer
pClpP-tev-His	Ap^R^, pET22b(+) expressing ClpP-tev-His, in which ClpP protein is fused with a TEV protease cleavage site and a His-tag in its C-terminal	Robert T. Sauer
pClpP_S__111__A_-tev-His	Ap^R^, pET22b(+) expressing ClpP-tev-His with ClpP S111A substitution	This study
pClpP_H__136__A_-tev-His	Ap^R^, pET22b(+) expressing ClpP-tev-His with ClpP H136A substitution	This study
pClpP_D__185__A_-tev-His	Ap^R^, pET22b(+) expressing ClpP-tev-His with ClpP D185A substitution	This study

### Plasmid Construction

All plasmids (Table 1) were confirmed by sequencing unless otherwise indicated. The complete list of primers used in this study is in Table 2. Plasmid pNptII was created by ligating the *Xho*I/*Bam*HI-digested *nptII* PCR product into the same restriction sites of pRL662. The plasmid was transformed into DH10B, and the resulting strain was designated as EML5395. The pRL-*rpsL*, pRL-*galK*, pRL-*nupG*, and pRL-*rpsL*^Str^ were created by ligating the *Xho*I/*Xba*I-digested PCR product into the same restriction sites of pRL662. The plasmid was transformed into DH10B, and the resulting strain was designated as EML5389, EML5390, EML5391, and EML5392. Plasmids pClpP-HA and pClpA-HA were created by ligating *Sac*I/*Pst*I-digested PCR products (*clpP* and *clpA* from BW25113 wild type without the stop codon, respectively) into pTrc200HA. The pClpP_S__111__A_-HA was created by amplifying fragments using pTRC99C-F plus ClpP-S111A-rv and pTRC99C-R plus ClpP-S111A-fw as primers. The two fragments were then merged and amplified by PCR-Splicing by Overlapping Extension (SOEing) (Heckman and Pease, 2007). The resulting full-length *clpP*-containing fragment was digested by *Sac*I/*Pst*I and then ligated into pTrc200HA. All other pClpP-HA plasmids with a mutated form of ClpP were created similarly. The plasmid constructs ClpX (ClpX-ΔN-ter), wild-type ClpP-tev-His, and green fluorescent protein (GFP)-ssrA were a kind gift from Dr. Robert T. Sauer (MIT, Cambridge, United States). Site-directed mutagenesis was performed to generate the ClpP variants. All plasmids of pClpP-tev-His with a mutated *clpP* gene was constructed similar to that of pClpP_S__111__A_-HA mentioned above with the differences below: Primer T7 was used instead of pTRC99C-F, and primer T7T was used instead of pTRC99C-R, and the restriction sites used were *Xba*I/*Xho*I.

**TABLE 2:** Primer information.

Primer	Sequence (5′–3′)^a^	Plasmids
T7	TAATACGACTCACTATAGGG	pET22b(+)
T7T	GCTAGTTATTGCTCAGCGG	
pTRC99C-F	TTGCGCCGACATCATAAC	pTrc200HA
pTRC99C-R	CTGCGTTCTGATTTAATCTG	
rpsL-fw	AAAAACTCGAGGCAAAAGCTAAAACCAGGA	(1) pRL-*rpsL*
rpsL-rv	AAAAATCTAGACTTACTTAACGGAGAACCA	(2) pRL-rpsL^Str^
galK-fw	AAAAACTCGAGCAGTCAGCGATATCCATT	pRL-galk
galK-rv	AAAAATCTAGAGCAAAGTTAACAGTCGGT	
nupG-fw	AAAAACTCGAGTCAAACACTCATCCGCAT	pRL-nupG
nupG-rv	AAAAATCTAGACCCGTTTTTCTTTGCGTAA	
NptII-fw-*Xho*I	AAAAACTCGAGAGACTGGGCGGTTTTATGGA	pNptII
NptII-rv-*Hin*dIII	AAAAAAAGCTTCTCTAGCGAACCCCAGAGTC	
ClpP-*Sac*I-fw	AAAAAGAGCTCATGTCATACAGCGGCGAACGAGATAAC	pClpP-HA
ClpP-*Pst*I-rv	AAAAACTGCAGATTACGATGGGTCAGAATCGAATCGAC	
ClpA-*Sac*I-fw	AAAAAGAGCTCATGCTCAATCAAGAACTGGAACTCAGTTT	pClpA-HA
ClpA-*Pst*I-rv	AAAAACTGCAGATGCGCTGCTTCCGCCTTGTGCTTT	
ClpP-S111A-fw	TGTATGGGCCAGGCGGCC**GCG**ATGGGCGCTTTCTTGCTG	(1) pClpP_S__111__A_-HA
ClpP-S111A-rv	CAGCAAGAAAGCGCCCAT**CGC**GGCCGCCTGGCCCATACA	(2) pClpP_S__111__A_-tev-His
ClpP-H136A-fw	AATTCGCGCGTGATGATT**GCC**CAACCGTTGGGCGGCTAC	(1) pClpP_H__136__A_-HA
ClpP-H136A-rv	GTAGCCGCCCAACGGTTG**GGC**AATCATCACGCGCGAATT	(2) pClpP_H__136__A_-tev-His
ClpP-D185A-fw	GAACGTGATACCGAGCGC**GCT**CGCTTCCTTTCCGCCCCT	(1) pClpP_D__185__A_-HA
ClpP-D185A-rv	AGGGGCGGAAAGGAAGCG**AGC**GCGCTCGGTATCACGTTC	(2) pClpP_D__185__A_-tev-His
ClpP-R26A-fw	GTCATTGAACAGACCTCA**GCC**GGTGAGCGCTCTTTTGAT	pClpP_R__26__A_-HA
ClpP-R26A-rv	ATCAAAAGAGCGCTCACC**GGC**TGAGGTCTGTTCAATGAC	
ClpP-D32A-fw	CGCGGTGAGCGCTCTTTT**GCT**ATCTATTCTCGTCTACTT	pClpP_D__32__A_-HA
ClpP-D32A-rv	AAGTAGACGAGAATAGAT**AGC**AAAAGAGCGCTCACCGCG	

*^a^Restriction enzyme sites are underlined, and mutated sequences are indicated in bold type.*

### Interbacterial Competition Assay

The optical densities of the cultured *A. tumefaciens* and *E. coli* were measured and adjusted to OD_600_ equals to 3.0 in 0.9% NaCl (w/v). The recipient *E. coli* cells were then further diluted to OD_600_ equals to 0.3 or 0.1, depending on the need of the assay. Afterward, the attacker and the recipient cultures were mixed in equal volume to make the attacker: recipient ratio 10:1 or 30:1, respectively. Ten microliters of the mixed bacterial culture was then spotted onto *Agrobacterium* Kill-triggering medium (AK medium, 3 g of K_2_HPO_4_, 1 g of NaH_2_PO_4_, 1 g of NH_4_Cl, 0.15 g of KCl, and 9.76 g of MES, pH 5.5), solidified by 2% (w/v) agar, and then air-dried to enable contact-dependent competition. The competition plates were cultured at 25°C for 16 h. After the competition, bacteria were recovered using a loop and resuspended into 500 μl of 0.9% NaCl. The recovered bacterial suspension was then serially diluted and plated onto LB supplemented with spectinomycin to select recipient *E. coli* cells. After overnight culture at 37°C, the recovered colony formation unit (cfu) was counted and recorded. The T6SS-dependent susceptibility index (SI) was defined as the logarithm of the recovered *E. coli* cfu co-cultured with Δ*tssL* subtracted by that co-cultured with wild-type *A. tumefaciens*.

### The High-Throughput Interbacterial Competition Platform

Pipetting steps of the screening platform were performed by the pipetting robot EzMate401 (Arise Biotech, Taiwan) unless otherwise specified. Fifty microliters of the cultured attacker *A. tumefaciens* was pelleted using 8,000 × *g* for 10 min at 15°C. After the medium was removed, the pellet was washed twice using 0.9% NaCl (w/v) and then adjusted to OD_600_ equals to 3.0. The OD_600_-adjusted attacker cells were then dispensed as 300 μl into each well of a 2.2-ml Deepwell microplate (Basic Life, Taiwan). Each well was then added with 10 μl of the cultured recipient *E. coli* mutants and mixed well to make the attacker:target at 30:1 (v/v). After being mixed, the bacterial mixture was then added onto the competition plate. The competition plate was made by 25 ml of the AK medium with 2% (w/v) agarose solidified in a 96-well lid. The competition plate was then cultured at 25°C for 16 h before recovery. The recovery was performed by stamping a 96-well plate replicator to the competition spots followed by suspending the bacterial cells to a 96-well plate containing 200 μl of 0.9% NaCl in each well. After being mixed, 10 μl of the recovered bacterial suspension was spotted onto LB agar supplemented with kanamycin made in a 96-well lid, cultured at 37°C overnight, and then was observed. In the first screening, only *A. tumefaciens* C58 wild type was used as the attacker. In the second screening, both wild type and Δ*tssL* were used as the attackers. For the groups co-cultured with *A. tumefaciens* C58 wild type, the recovery suspension was either undiluted or diluted to 5 and 25 times before being spotted onto LB agar with kanamycin plate. For the groups co-cultured with *A. tumefaciens* C58 Δ*tssL*, the recovery suspensions were either undiluted or diluted to 10 and 100 times before spotted onto LB agar with kanamycin plate. At each stage, the *E. coli* mutants that formed multiple colonies were identified as the candidates.

### Protein Production and Purification

*Escherichia coli* BL21(DE3) was used as a host to produce all proteins of interests. Cells were cultured in LB medium supplemented with appropriate antibiotics in 1-L flask. When OD_600_ reached 0.6, the bacterial culture was cooled to 16°C, and IPTG was added (final concentration of 0.5 mM) for the overexpression of the protein. The cells were further allowed to grow for 16 h, followed by centrifugation to pellet them and then resuspended in lysis buffer (50 mM of Tris, pH 8.0, 300 mM of NaCl, 1% Triton X-100, 10 mM of beta-mercaptoethanol, 1 mM of DTT, and 10% glycerol). The cells were lysed by sonication at 4°C (amplitude 10 for 5 s, followed by 15-s breaks; total sonication time was 6 min) (PRO Scientific, United States). The lysates were centrifuged at 20,000 rpm for 30 min at 4°C. The supernatants were collected and loaded onto Ni-NTA column (GE Healthcare, United States) equilibrated with wash buffer (50 mM of Tris, pH 8.0, and 300 mM of NaCl) and eluted by 6 ml of wash buffer containing 250 mM of imidazole. The eluted fractions of the protein were further subjected to size-exclusion chromatography (SEC) by Superdex 200, 16/60 column (GE Life Sciences, United States) in buffer containing 50 mM of Tris, pH 7.5, 100 mM of KCl, 25 mM of MgCl_2_, 1 mM of DTT, and 10% glycerol. The protein purity was confirmed on 12% sodium dodecyl sulfate–polyacrylamide gel electrophoresis (SDS-PAGE). The samples were flash-frozen and stored in −80°C until further use.

### Protein Degradation Assay

Green fluorescent protein (GFP) fluorescence based-degradation assays were carried out in Protein Degradation (PD) buffer (25 mM of HEPES, pH 7.5, 100 mM of KCl, 25 mM of MgCl_2_, 1 mM of DTT, and 10% glycerol) containing 3 μM of GFP-ssrA as substrate and ATP regeneration system (16 mM of creatine phosphatase and 0.32 mg/ml of creatine kinase) as described previously (Sriramoju et al., 2018). In brief, 0.1 μM of ClpX_6_ and 0.3 μM of ClpP_14_ or its variants were mixed at 30°C and allowed to stand for 2 min. The protein degradation reaction was started by addition of ATP to a final concentration of 5 mM. The changes in the fluorescence were measured at 511 nm with an excitation wavelength at 467 nm in a 96-well format using Infinite M1000 PRO plate reader (Tecan, Switzerland).

### Sodium Dodecyl Sulfate–Polyacrylamide Gel Electrophoresis and Western Blot Analysis

The Δ*clpP*:kan *E. coli* strains harboring appropriate plasmid were grown as the same procedure indicated in the *Interbacterial Competition Assay*. Cells were adjusted to OD_600_ of 5.0, collected at 5,000 × *g* for 5 min, and directly resuspended in 1 × SDS sample buffer. The samples were incubated at 96°C for 10 min and then analyzed by SDS-PAGE. Protein samples separated by SDS-PAGE were transferred to an Immobilon-P membrane (Merck Millipore, United States). The monoclonal anti-HA was used at a dilution of 1:10,000 (Yao-Hong Biotech Inc., Taiwan), and the goat–anti-rabbit conjugated to horseradish peroxidase secondary antibody was used at a dilution of 1:10,000 (GeneTex, Taiwan). The Western Lightning ECL Pro (PerkinElmer Life Sciences, United States) was used for color development and visualized by BioSpectrum 600 Imaging System (UVP, United States).

### Statistical Analysis and Figure Production

Statistical analyses and figure production were performed using the R program (version 3.5.1) (R Core Team, 2018) and RStudio (version 1.1.456) (RStudio Team, 2015). R packages plyr (version 1.8.4) (Wickham, 2011) and multcompView (version 0.1-7) (Graves et al., 2015) were used for statistical analyses. Figures were produced using the R packages ggplot2 (version 3.0.0) (Wickham, 2016), Hmisc (version 4.1-1) (Harrell et al., 2018), and ggpubr (version 0.2) (Kassambara, 2018). Student’s *t*-test, one-way analysis of variance (one-way ANOVA), and Tukey’s honestly significant difference test (Tukey HSD test), in which significant difference threshold set as 0.05, were used in all cases.

## Results

### The *Agrobacterium tumefaciens* T6SS Killing Outcome Differs Between Different *Escherichia coli* Strains

**FIGURE 1:**
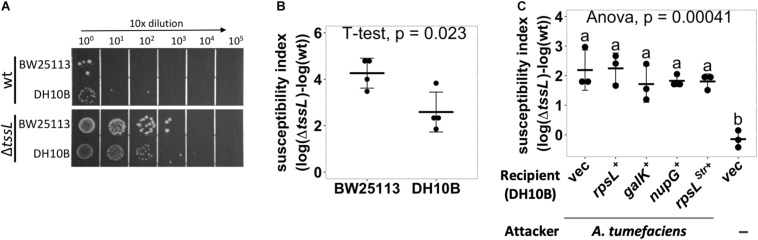
*Agrobacterium tumefaciens* type VI secretion system (T6SS)-dependent antibacterial activity against *Escherichia coli* strains. **(A,B)** *A. tumefaciens* T6SS antibacterial activity against *E. coli* strains DH10B and BW25113. *A. tumefaciens* was co-cultured at a ratio of 30:1 with *E. coli* DH10B or BW25113, both *E. coli* strains harboring vector pRL662, on *Agrobacterium* Kill (AK) agar medium for 16 h. The bacterial mixtures were serially diluted and spotted **(A)** or quantified by counting cfu **(B)** on gentamicin-containing lysogeny broth (LB) agar plates to selectively recover *E. coli*. **(C)** *E. coli* DH10B was complemented by either vector only (vec) or derivative expressing *rpsL*, *galK*, *nupG*, or *rpsL^Str^ in trans* before being subjected to *A. tumefaciens* T6SS-dependent antibacterial activity assay as described in **(B)**. Susceptibility index (SI) was defined as the subtraction difference of the recovery log(cfu) of that attacked by Δ*tssL* to that attacked by wild-type *A. tumefaciens* C58. Data are mean ± SD of three independent experiments calculated by *t*-test with *P* < 0.05 for statistical significance **(B)** or single-factor analysis of variance (ANOVA) and Tukey honestly significant difference (HSD), in which two groups with significant differences are indicated with different letters (a and b) **(C)**.

Using an optimized competition condition (AK medium agar that contains basic minerals at pH 5.5), we noticed that when co-cultured with wild-type *A. tumefaciens* C58, the recovered colony-forming unit (cfu) of *E. coli* BW25113 was always lower than that of DH10B (Figure 1A). Meanwhile, the recovered cfu of both *E. coli* strains was the same when co-cultured with Δ*tssL A. tumefaciens* C58 (hereafter referred to Δ*tssL*), a T6SS secretion-deficient mutant (Figure 1A). For more intuitive readout, we introduced T6SS-dependent SI, which reflects the strength of the T6SS killing. The SI was defined as the logarithm of the recovered *E. coli* cfu co-cultured with Δ*tssL* subtracted by that co-cultured with wild type. The mean SI between *A. tumefaciens* and BW25113 was significantly higher than that of between *A. tumefaciens* and DH10B with a *P*-value of 0.02 (*T* ≤ *t*, two-tailed, Figure 1B). This result suggests that some genetic factors of BW25113 may enhance the *A. tumefaciens* C58 killing outcome in a T6SS-dependent manner.

We tested whether the genes that are functional in BW25113 but not in DH10B could be the cause of the higher SI in BW25113. The *galK* and *nupG* genes are functional in BW25113 but are pseudogenes in DH10B. The *rpsL* has a mutation in DH10B (*rpsL*^Str^), which renders the strain resistant to streptomycin, but not in BW25113. The *rpsL*, *galK*, or *nupG* gene from BW25113 was cloned into pRL662 and expressed by constitutive *lacZ* promoter in DH10B as a recipient for a T6SS interbacterial competition assay (Figure 1C). The DH10B expressing the *rpsL*^Str^ (overexpressing *rpsL*^Str^) was also included. The DH10B harboring empty vector (vec) served as a negative control. A group without attacker was also included to monitor whether the decrease in cfu after the competition solely comes from co-culture with *A. tumefaciens* attacker. The SIs were not significantly different between DH10B and any of the complemented groups, and each had an SI mean of about 2 (Figure 1C). The above approach was not able to identify the genetic factors that contributed to the enhanced resistance in DH10B, which may imply that precise control of transgene expression or multiple complementation would be required. Therefore, we developed a high-throughput screening method to identify the individual genes that contribute to the enhanced susceptibility of BW25113.

### Establishment of a High-Throughput Interbacterial Competition Platform to Identify Recipient *Escherichia coli* Mutants With Less Susceptibility to *Agrobacterium tumefaciens* C58 T6SS Killing

**FIGURE 2:**
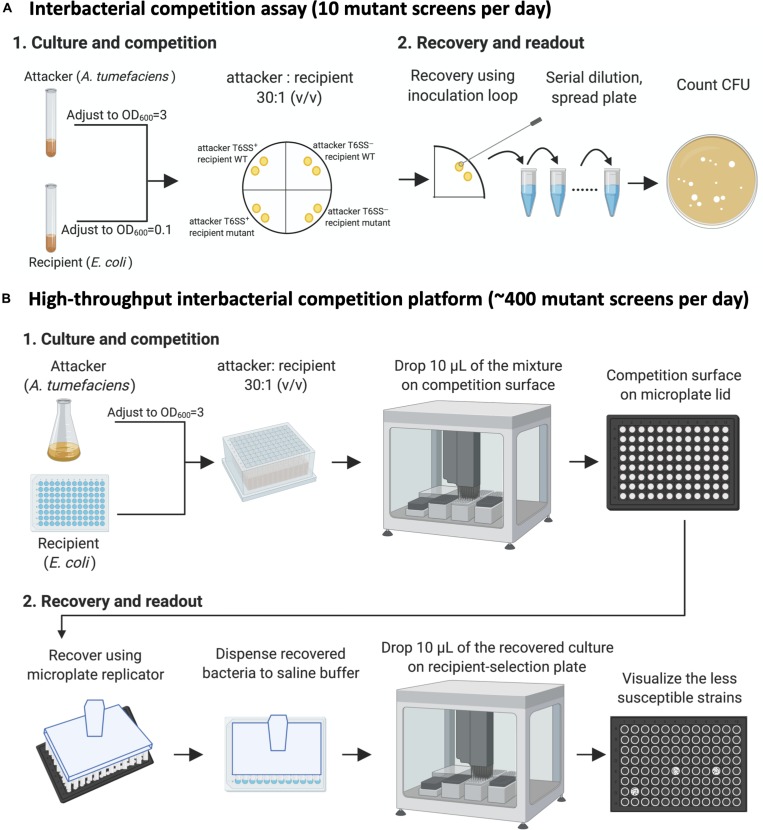
The high-throughput interbacterial competition platform. **(A)** Interbacterial competition assay. Cultured attacker *Agrobacterium tumefaciens* and recipient *Escherichia coli* were mixed and then spotted on the *Agrobacterium* Kill (AK) agar medium to allow interbacterial competition for 16 h at 25°C followed by recovery of mixed cultures, serially diluted, and then spread onto lysogeny broth (LB) plate supplemented with appropriate antibiotics to select for recipient cells. **(B)** High-throughput interbacterial competition screening platform. Recipient cells were grown and mixed with attacker *A. tumefaciens* in a 96-well plate. The bacterial mixture was dropped onto the AK agar medium competition surface using an automated pipetting system. The competition surface was made on a microplate lid. Recovery was performed using microplate replicator. The candidates are the strains that show multiple colonies grown after recovery as opposed to wild-type controls and most strains with no or few colonies. This high-throughput *A. tumefaciens* type VI secretion system (T6SS) killing platform enables ∼400 mutant screens per day. This figure was created with BioRender (https://biorender.com/).

We decided to screen the BW25113 single-gene mutant library (Keio collection from NBRP [NIG, Japan]: *E. coli*) for strains with less susceptibility to *A. tumefaciens* T6SS-mediated killing. An interbacterial competition assay starts from mixing the attacker and the recipient cells, followed by counting the recovered recipient *E. coli* on selective media (Figure 2A). This protocol only allowed screening of 10 mutants per day, which was not efficient enough for screening 3,909 strains of the Keio library.

Therefore, we developed a high-throughput interbacterial competition platform that enables 96 population-level, interbacterial competition simultaneously (Figure 2B). The recipient Keio *E. coli* strains were cultured in the 96-well, and the attacker *A. tumefaciens* was cultured in a flask. After the culture, the attacker was adjusted to OD_600_ equals to 3.0 and then dispensed to a 2.2-ml deep-well plate. The recipient cells were added into the attacker-containing plates in a volume ratio of 30 to 1. Ten microliters of the attacker-recipient mixtures were dropped on the competition surface made by agar solidified on a 96-well lid. A microplate replicator was used to stamp on the competition spots to recover the bacterial cells of each competition group. The recovered bacteria were suspended in the saline buffer (0.9% NaCl), mixed, and then spotted on the recipient-selection surface made by agar solidified on a microplate lid. The competition condition was set at the strength that enables *A. tumefaciens* to kill almost all BW25113 wild-type recipients so that only a few or no cells would survive. This setup made recognizing the resistant mutants simple – the ones with the multiple colonies are the candidates (Figure 2B).

All the 3,909 strains in the Keio were screened using *A. tumefaciens* C58 wild type as the attacker. In each screening, at least two wild type *E. coli* BW25113 replicates were incorporated and screened in parallel as parental controls. The Keio mutants that formed colonies in this stage were selected, and 196 strains showed enhanced resistant to *A. tumefaciens* C58 attack. The 196 strains were subjected to second screening using both wild type and Δ*tssL* as the attackers. At this stage, we incorporated a grading system: Grade I mutants were at least 25 times less susceptible to C58 T6SS-dependent killing, whereas grade II mutants were at least 10 times less susceptible. Six grade I mutants and 10 grade II mutants were identified.

### Confirmation of the *Escherichia coli* Mutants With Less Susceptibility to *Agrobacterium tumefaciens* C58 Type VI Secretion System Killing

The enhanced resistance of the six grade I mutants were further verified by an interbacterial competition assay and by complementation tests. For complementation, wild-type genes from BW25113 were cloned into plasmid pTrc200HA plasmid and expressed by *trc* promoter. Five out of six showed lower susceptibility to *A. tumefaciens* T6SS attack than that of BW25113 wild type (Table 3 and Supplementary Figure S1). These are *clpP*, *gltA*, *ydhS*, *ydaE*, and *cbpA* mutants. The *yeaX* mutant, on the other hand, did not differ when compared with the wild type. The *cbpA* mutant showed a milder phenotype and could not be complemented *in trans* under the condition tested (Table 3 and Supplementary Figure S1). As *cbpA* is the first gene in its operon, the failure in complementation could be due to the requirement of other gene(s) in the operon. Nevertheless, the verification performed above showed that the high-throughput interbacterial competition platform was reliable in identifying the recipient genetic factors that participate in T6SS killing.

**TABLE 3:** *Escherichia coli* strains that showed reduced susceptibility to *Agrobacterium tumefaciens* T6SS attack.

No.	Resource	Disrupted	Gene products affected by	Reduced	Trans
	(JW ID)	gene	kanamycin cassette insertion^a^	susceptibility^b^	complementation^c^
1	JS0427	*clpP*	ClpAXP, ClpXP, ClpAP	O	O
2	JW0710	*gltA*	citrate synthase	O	O
3	JW1658	*ydhS*	FAD/NAD(P) binding domain-containing protein YdhS	O	O
4	JW1346	*ydaE*	Rac prophage; zinc-binding protein	O	Δ
5	JW0985	*cbpA*	Curved DNA-binding protein	O	X
6	JW1792	*yeaX*	Carnitine monooxygenase	X	n.d.

*n.d., not determined. ^*a*^Gene products information was obtained from the EcoCyc database (Keseler et al., 2016). ^*b*^Mutant strains with reduced susceptibility index (SI) and showed significant difference under *P* < 0.05 was labeled as O; those with no significant difference to that of wild type were labeled in X. ^*c*^Plasmid-born gene that can fully complement the disrupted gene is labeled in O, partially complemented is labeled in Δ, and cannot be complemented is labeled in X.*

### The ClpP Protein Plays a Role in Enhancing Susceptibility to *Agrobacterium tumefaciens* Type VI Secretion System Killing

**FIGURE 3:**
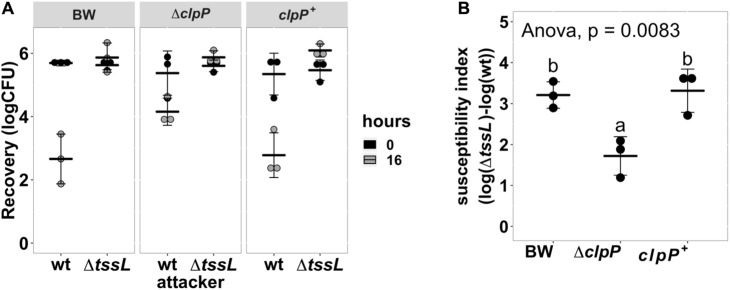
*Agrobacterium tumefaciens* susceptibility to type VI secretion system (T6SS)-dependent antibacterial activity was reduced in *Escherichia coli clpP:kan* and can be fully complemented *in trans*. **(A)** Recovery of surviving *E. coli* cells at 0 h and 16 h after being co-cultured with either *A. tumefaciens* wild type C58 (wt) or Δ*tssL* at a ratio of 30:1. **(B)** The susceptibility index (SI) of *E. coli* BW25113 wild type (BW), Δ*clpP*, and Δ*clpP* complemented with *clpP* expressed on plasmid (*clpP*^+^) was calculated from the recovery rate shown in **(A)**. Statistical analysis involved single-factor analysis of variance (ANOVA) and Tukey honestly significant difference (HSD). Data are mean ± SD of three independent experiments, and two groups with significant differences are indicated with different letters (a and b) (*P* < 0.05 for statistical significance).

Because known recipient cell factors affecting antibacterial activity are often conserved components, we selected Δ*clpP*:kan (labeled as Δ*clpP*) for further studies. ClpP is a highly conserved, housekeeping AAA^+^ serine protease that exists in prokaryotes, plastids, and mitochondria (Alexopoulos et al., 2012; Bhandari et al., 2018; Mahmoud and Chien, 2018). We performed a quantitative interbacterial competition assay using *A. tumefaciens* as the attacker and the BW25113 wild type, Δ*clpP*, or complemented strain *clpP*^+^ as the recipient cells (Figure 3A). The initial cfu of the *E. coli* at 0 h was about 10^6^ in all groups (one-way ANOVA with *P* = 0.88), indicating that any *E. coli* cfu difference at 16 h was not due to initial bacteria titer difference. The cfu among different recipient *E. coli* strains was not significantly different at 16 h when using *A. tumefaciens* Δ*tssL* (one-way ANOVA with *P* = 0.67), indicating that co-culture with T6SS-deficient strain will not cause recipient titer to differ. On the other hand, the recovered cfu of Δ*clpP* was about 10^4^, whereas it was about 5 × 10^2^ in BW25113 wild type and in *clpP*^+^ after 16-h competition using wild-type *A. tumefaciens* (Figure 3A). The mean SI of the BW25113 wild type to *A. tumefaciens* C58 is significantly higher than that of Δ*clpP* (one-way ANOVA with *P* = 0.02, Figure 3B). The less susceptible phenotype of the Δ*clpP* can be fully complemented *in trans* (*clpP*^+^) (*P* = 0.96 compared with BW25113 wild type). These results confirmed that *clpP* contributes to enhancing susceptibility to T6SS antibacterial activity of *A. tumefaciens* C58.

### Effects of ClpP Catalytic Variants in Enhancing *Agrobacterium tumefaciens* Type VI Secretion System Antibacterial Activity and Protease Activity

**FIGURE 4:**
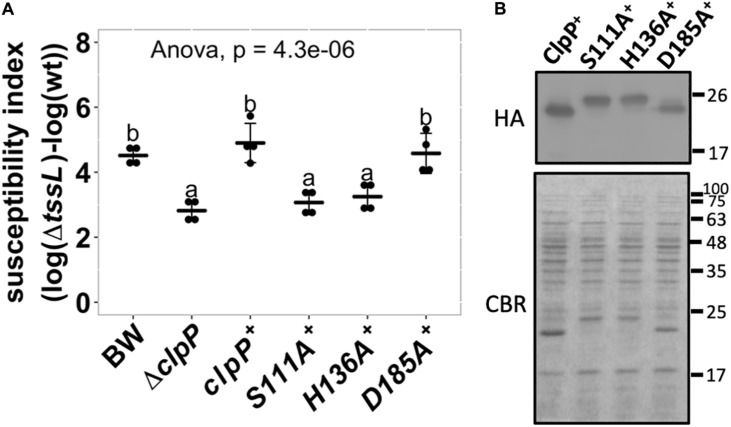
Effects of ClpP protease catalytic variants in enhancing *Agrobacterium tumefaciens* type VI secretion system (T6SS) antibacterial activity. **(A)** The susceptibility index calculated from *A. tumefaciens* interbacterial activity assay against *Escherichia coli*. The *A. tumefaciens* C58 wild-type or Δ*tssL* were co-cultured at a ratio of 10:1 with *E. coli* BW25113 wild type (BW), Δ*clpP*, and Δ*clpP* complemented with *clpP* and its variants expressed on plasmid. The complemented *clpP* strains were either wild type (clpP^+^) or catalytic variants ClpP_S111A_ (S111A^+^), ClpP_H136A_ (H136A^+^), and ClpP_D185A_ (D185A^+^), with C-terminus HA-tag. The susceptibility index (SI) of each *E. coli* was calculated from the logarithm recovery rate of the Δ*tssL* co-cultured group minus that of the wild-type co-cultured group. Data are mean ± SD of four biological replicates from two independent experiments. Statistical analysis involved single-factor analysis of variance (ANOVA) and Tukey honestly significant difference (HSD) with *P* < 0.05 for statistical significance. Two groups with significant differences are indicated with different letters (a and b). **(B)** The ClpP protein levels of the Δ*clpP* complemented strains used in **(A)**. The ClpP-expressing *E. coli* strains were cultured at the same condition used in interbacterial competition assay. Instead of co-culture with *A. tumefaciens*, protein samples were collected, normalized, and subjected to Western blot analysis of ClpP:HA and its variants. Representative result of three independent experiments is shown.

A functional ClpP complex consists of a tetradodecameric ClpP (ClpP_14_) and its associated AAA^+^ ATPase substrate-recognizing partner ClpA or ClpX, both in a hexameric form (Olivares et al., 2015). The protease catalytic triad of the *E. coli* ClpP is composed of S111, H136, and D185 (counted from the Met1) (Maurizi et al., 1990; Wang et al., 1997). We tested whether the ClpP protease is essential in enhancing *E. coli* susceptibility to *A. tumefaciens* C58 T6SS attack. *E. coli*Δ*clpP* complemented with pTrc200HA expressing either wild-type or catalytic variants ClpP S111A, H136A, and D185A was used as a recipient strain. All ClpP variants contain a C-terminal HA tag. Two of the catalytic variants, S111A^+^ and H136A^+^, failed to complement, whereas surprisingly, the third catalytic variant, D185A^+^, can fully complement the phenotype (Figure 4A). The difference of ClpP catalytic variants to complement Δ*clpP* was not due to their protein-expression level as determined by Western blot (Figure 4B). The protein migration of the ClpP_S__111__A_ and ClpP_H__136__A_ was slower than that of the ClpP_wt_ and ClpP_D__185__A_ owing to their inability to remove the N-terminal propeptide (1–14 amino acids) as in ClpP_wt_ and ClpP_D__185__A_ (Maurizi et al., 1990; Bewley et al., 2006).

**FIGURE 5:**
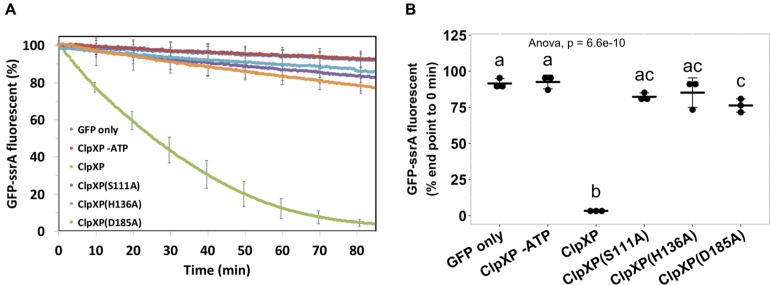
Protease activity assay of the ClpP and its catalytic variants. The wild-type ClpP and its catalytic variants were each pre-assembled with ClpX followed by providing its substrate, the ssrA-tagged green fluorescent protein (GFP). The GFP fluorescent signals were monitored **(A)** over time, and **(B)** statistical analysis was measured at the end of the assay. Statistical analysis involved single-factor analysis of variance (ANOVA) and Tukey honestly significant difference (HSD) with *P* < 0.05 for statistical significance. Two groups with significant differences are indicated with different letters (a and b). Data are mean ± SD of three biological replicates from one representative result of at least two independent experiments.

As ClpP_D__185__A_ was able to complement the phenotype, we further investigated the ClpP protease activity of the above ClpP variants by a widely adopted ClpP protein degradation assay using GFP-ssrA as the model substrate. Loss of GFP fluorescence is used as a reporter to monitor substrate degradation by ClpXP as a function of time (Weber-Ban et al., 1999; Sriramoju et al., 2018). The results showed that over time, wild-type ClpP effectively degraded GFP-ssrA with a half-life of about 30 min (Figure 5A). Meanwhile, less than a 10% decrease of the GFP-ssrA signal was observed in GFP-ssrA only and wild type without ATP groups, both served as negative controls. The decreasing rates of the GFP-ssrA fluorescence of ClpP_S__111__A_, ClpP_H__136__A_, and ClpP_D__185__A_ were significantly slower than those of ClpP_WT_ and showed no significant difference among the three variants at the end of the test (Figures 5A,B). Although ClpP_D__185__A_ showed no statistically difference in GFP-ssrA degradation compared with ClpP_S__111__A_ and ClpP_H__136__A_ at the final time point, it showed significantly lower GFP-ssrA signal to that of the negative control groups (Figure 5B).

### The ClpP-Associated AAA^+^ ATPase ClpA but Not ClpX Is Involved in Enhancing Susceptibility to *Agrobacterium tumefaciens* Type VI Secretion System Activity

**FIGURE 6:**
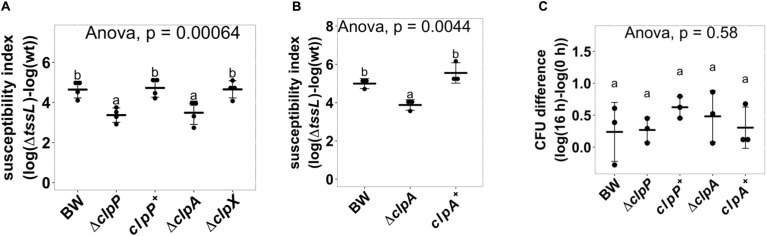
ClpP associated AAA^+^ ATPase ClpA but not ClpX is involved in enhancing *Agrobacterium tumefaciens* type VI secretion system (T6SS) antibacterial activity. **(A)** *A. tumefaciens* T6SS antibacterial activity against *Escherichia coli*Δ*clpP* and its complement strain, Δ*clpA* and Δ*clpX*. The *A. tumefaciens* and the *E. coli* were co-cultured at a ratio of 10:1 on *Agrobacterium* Kill (AK) agar medium for 16 h. Afterward, the recovery of *E. coli* strains was quantified, and the susceptibility index was calculated by subtracting the difference of the recovered log(cfu) of that attacked by Δ*tssL* to that by wild-type *A. tumefaciens* C58. **(B)** *A. tumefaciens* T6SS antibacterial activity assay and the susceptibility index were performed as described in **(A)** using *E. coli* wild type (BW), Δ*clpA*, and Δ*clpA* complemented with *clpA* expressed on plasmid (*clpA*^+^). **(C)** Growth of *E. coli* when co-culturing with the Δ*tssL A. tumefaciens*. Data in **(A**–**C)** are mean ± SD of at least three independent experiments. Statistical analysis involved single-factor analysis of variance (ANOVA) and Tukey honestly significant difference (HSD) with *P* < 0.05 for statistical significance. Two groups with significant differences are indicated with different letters (a and b).

**FIGURE 7:**
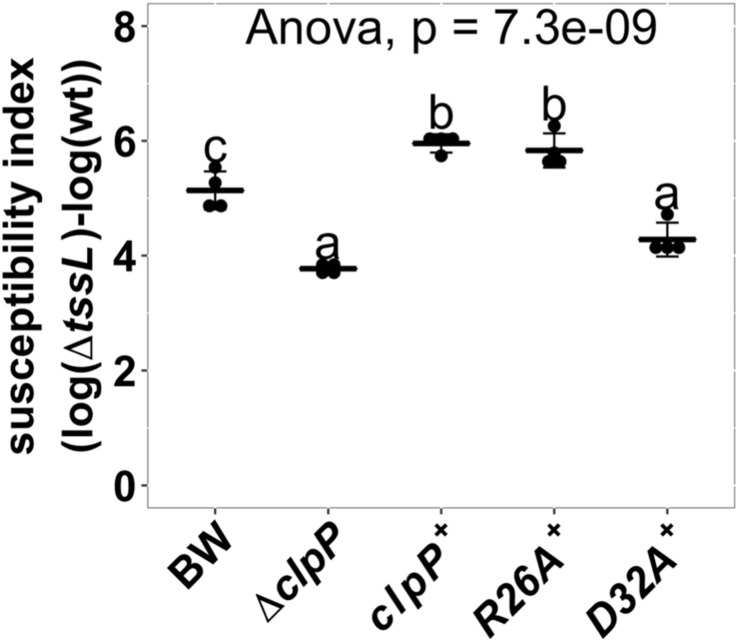
Effects of ClpP variants impaired with ClpA-binding ability in enhancing *Agrobacterium tumefaciens* type VI secretion system (T6SS) antibacterial activity. Interbacterial competition assay between *A. tumefaciens* and *Escherichia coli* wild type, Δ*clpP*, and Δ*clpP* complement strains expressing wild-type ClpP (*clpP*^+^), ClpAP complex formation mutants ClpP_R26A_ (R26A^+^), and ClpP_D32A_ (D32A^+^). The ClpAP complex forming ability is half than that of wild-type ClpP in ClpP_R26A_ and is completely lost in ClpP_D32A_ (Bewley et al., 2006). The T6SS killing data are mean ± SD of four biological replicates from two independent experiments. Statistical analysis involved single-factor analysis of variance (ANOVA) and Tukey honestly significant difference (HSD) with *P* < 0.05 for statistical significance. Two groups with significant differences are indicated with different letters (a and b).

ClpP is a protein protease dependent on other adapter proteins such as ClpA and ClpX for substrate recognition (Maurizi, 1991; Gottesman et al., 1998). Therefore, we next determined whether the resistant phenotype of Δ*clpP* is mediated by ClpA or ClpX through the interbacterial competition assay of the deletion mutants Δ*clpA*:kan (hereafter referred to as Δ*clpA*) and Δ*clpX*:kan (hereafter referred to as Δ*clpX*) as recipients. SI demonstrates that Δ*clpA* was less susceptible to *A. tumefaciens* T6SS killing than BW25113 wild-type (*P* = 0.02), whereas Δ*clpX* was similar to BW25113 wild type (*P* = 1.00) (Figure 6A). The decreased *A. tumefaciens* T6SS killing phenotype of Δ*clpA* was fully complemented *in trans* (Figure 6B). No difference could be detected among the growth of the BW25113 wild-type, Δ*clpA*, Δ*clpP*, and their respective complemented strains when co-cultured with Δ*tssL* (*P* = 0.58) (Figure 6C). Therefore, the killing outcome is caused by *Agrobacterium* T6SS-mediated interbacterial competition rather than the growth rate of the different recipient strains under the competition condition. This suggested that ClpA could be the adapter that interacts with ClpP leading to the enhanced susceptibility to T6SS attack in BW25113 wild type. In this case, the interaction between ClpA and ClpP should be required for enhancing *A. tumefaciens* T6SS killing. The interaction between ClpA and ClpP is well studied, and it has been demonstrated that the R26A and D32A variants of ClpP lose their ability to bind to ClpA by 50 and 100%, respectively (Bewley et al., 2006). Therefore, we complemented ClpP_R__26__A_ and ClpP_D__32__A_ in Δ*clpP* to determine whether the two variants could restore the susceptibility. The R26A^+^ was able to complement (*P* = 0.96, compared to ClpP^+^), whereas D32A^+^ failed to complement and showed no statistical difference in SI than that of Δ*clpP* (*P* < 10^–4^) (Figure 7). These results suggest that the phenotype observed in Δ*clpP* and in Δ*clpA* could be associated with ClpA–ClpP interaction. Because the retained N-terminal propeptide does not prevent ClpP–ClpA binding (Maurizi et al., 1990), the inability of unprocessed ClpP_S__111__A_ and ClpP_H__136__A_ in enhanced susceptibility is independent of ClpP–ClpA complex formation.

## Discussion

This study provides evidence that the genetic factors of the recipient cells play an important role in affecting the outcome of the T6SS antibacterial activity. The high throughput interbacterial competition platform developed in this study proved to be an effective method in identifying recipient factors that affect the outcome of *A. tumefaciens* T6SS antibacterial activity. Further exploration led to the confirmation of at least six genes (*clpP*, *clpA*, *gltA*, *ydhS*, *ydaE*, and *cbpA*) encoding known or putative cytoplasmic proteins (Keseler et al., 2016), whereas CbpA resides both in the cytoplasm and in the nucleoid (Orfanoudaki and Economou, 2014). None of these gene products were localized to the inner membrane, periplasm, outer membrane, or extracellular milieu. This result implies that the process affecting the outcome of *A. tumefaciens* T6SS killing to *E. coli* occurs in the cytoplasm, presumably after the injection of the T6SS puncturing apparatus. Previous studies have mainly focused on how attacker T6SS is regulated and sensed (Filloux and Sagfors, 2015; Alteri and Mobley, 2016; Hood et al., 2017). This study provides a new insight that recipient cell genes can also affect the T6SS killing outcome and that it could take place after the injection of the T6SS apparatus into the recipient cytoplasm.

Our data showed that ClpA but not ClpX, together with ClpP, contributes to the susceptibility of the recipient *E. coli* to *A. tumefaciens* T6SS killing. The *clpX* transcript level drops and fades 15 min after the onset of carbon starvation (Li et al., 2000), which is the condition used for our interbacterial competition. Thus, ClpX is probably not available to form the ClpXP complex during *Agrobacterium* T6SS attacks. The Δ*clpA* was indeed identified in the first screening but was accidentally misplaced and did not enter the second screening process. Therefore, Δ*clpA* did not appear in our final candidate list until we obtained the correct strain for confirmation. The results that the three catalytic variants ClpP_S__111__A_, ClpP_H__136__A_, and ClpP_D__185__A_ did not significantly differ in their ability to degrade GFP-ssrA substrate suggested that the protease activity may not be the leading cause in enhancing *A. tumefaciens* T6SS attack. On the other hand, unlike ClpP_S__111__A_ and ClpP_H__136__A_, which do not exhibit significant protease activity as compared with that of the negative controls, ClpP_D__185__A_ may possess weak protease activity, as the GFP-ssrA fluorescence level is significantly lower than that of the negative controls at the final time point. Thus, the involvement of the ClpP protease activity cannot be completely ruled out as the weak protease activity of ClpP_D__185__A_ may be sufficient to exhibit its function in enhancing *A. tumefaciens* T6SS attack. Of note, the ClpP protease activity monitored by the *in vitro* protease activity assay using either ClpX or ClpA as a protein unfoldase showed a highly similar pattern among 24 ClpP variants (Bewley et al., 2006). As this GFP-ssrA degradation assay is an *in vitro* system and that it is difficult to monitor the ClpP protease activity of the recipient under competition condition, the role of ClpP protease remains elusive.

Our data also suggest that ClpAP complex is required in enhancing recipient susceptibility during *A. tumefaciens* T6SS killing on the basis of the results that ClpP variant that loses its ability to form a complex with ClpA did not complement the phenotype whereas those with ClpA binding ability do. This implies that the ClpA–ClpP complex, rather than ClpP alone, is the cause of the enhanced susceptibility to T6SS attack. As ClpP allosterically activates the polypeptide translocation activity of ClpA (Miller et al., 2013), the necessity of the ClpAP complex may depend on the unfoldase activity of ClpA. The detailed mechanism on how recipient ClpAP is involved in T6SS susceptibility enhancement awaits further investigations. One promising future direction would be identifying the potential ClpA substrates and their effects on increasing susceptibility of T6SS attack.

Hijacking a highly conserved and essential molecule of the recipient cell to improve attacker fitness is not uncommon. The examples are CdiA-CT^EC93^ hijacking essential proteins BamA and AcrB, CdiA-CT^EC536^ hijacking the recipient CysK, and Ssp2 and Ssp4 hijacking recipient DsbA (Aoki et al., 2008; Diner et al., 2012; Mariano et al., 2018). The ClpP protease, on the other hand, is highly conserved in both prokaryotes and eukaryotic organelles like plastid and mitochondria (Culp and Wright, 2016; Moreno et al., 2017). The ClpP protease cooperates with different AAA^+^ ATPases in different organisms. It works with ClpA and ClpX in Gram-negative bacteria; with ClpC and ClpE in Gram-positive bacteria; with ClpC1, ClpC2, and ClpD in the chloroplast; and with ClpX in human mitochondria. In all these cases, the ClpP protease seems to play a central role in protein homeostasis. Dysfunction of the system can lead to severe developmental defects, a reduction in the pathogenicity, or lethality (Cole et al., 2015; Nishimura and Van Wijk, 2015; Bhandari et al., 2018). The current result suggests that the ClpP protease system could be another target hijacked by the T6SS attacker to improve its competitive advantage.

To our knowledge, the involvement of the ClpAP complex in enhancing the recipient’s susceptibility to *A. tumefaciens* T6SS activity has not been described in the contact-dependent competitor elimination systems in Gram-negative bacteria like T1SS, T4SS, CDI, and T6SS. It would be of interest to uncover how and what *A. tumefaciens* factors hijack this universal and highly conserved ClpP and its associated AAA^+^ ATPase substrate recognizing partner. The current finding provides additional evidence to support that T6SS can manipulate the essential and highly conserved molecules of recipient cells to achieve better inhibition of the performance (Russell et al., 2014). Elucidating the underlying molecular mechanisms of ClpAP and other recipient factors would be the next direction to understand further how genetic factors can affect the recipient susceptibility to the T6SS attacks.

## Data Availability Statement

All datasets generated for this study are included in the article/Supplementary Material.

## Author Contributions

H-HL, MY, and E-ML conceived and designed the experiments. H-HL performed most of the experiments. MS contributed to the protease activity assay. MS and S-TH provided the materials and tools for the protease activity assay. S-TH, C-TL, and E-ML supervised the execution of the experiments. H-HL and E-ML, with contributions from MY, MS, S-TH, and C-TL wrote the manuscript. All authors read and approved the final manuscript.

## Conflict of Interest

The authors declare that the research was conducted in the absence of any commercial or financial relationships that could be construed as a potential conflict of interest.
