# Supplementary material for: A High-Throughput Interbacterial Competition Screen Identifies ClpAP in Enhancing Recipient Susceptibility to Type VI Secretion System-Mediated Attack by Agrobacterium tumefaciens
Source: Front Microbiol. 2020 Feb 5;10:3077. doi: 10.3389/fmicb.2019.03077 (PMC7012810; doi:10.3389/fmicb.2019.03077)
Supplement: Supplementary file 1 [file Data_Sheet_1.PDF]

Supplementary Material

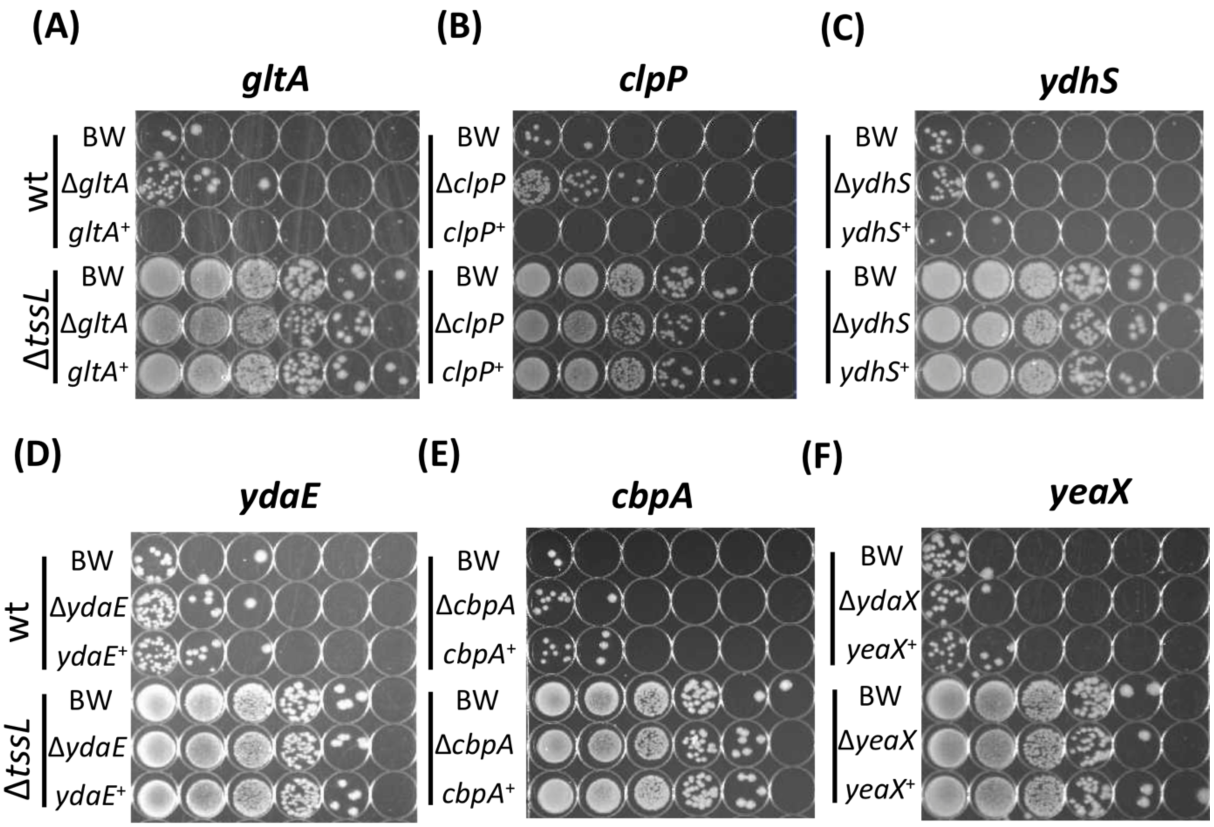

Supplementary Figure 1. Interbacterial competition assay between *A. tumefaciens* C58 and the *E. coli* candidates that were less susceptible to T6SS killing. The *A. tumefaciens* C58 wild-type or Δ*tssL* were co-cultured at a ratio of 30:1 with *E. coli* BW25113 wild type (BW), the Keio mutant strains, and complemented strains expressing the mutated genes *in trans*. The Keio strain used was **(A)** Δ*gltA*, **(B)** Δ*clpP*, **(C)** Δ*ydhS*, **(D)** Δ*ydaE*, **(E)** Δ*cbpA*, and **(F)** Δ*yeaX*. Data shown are its representative results; at least two independent experiments were performed in each group.
